# Cardiac Magnetic Resonance Imaging Detects Myocardial Abnormalities in Naturally Infected Dogs with Chronic Asymptomatic Chagas Disease

**DOI:** 10.3390/ani13081393

**Published:** 2023-04-18

**Authors:** Derek J. Matthews, Ryan C. Fries, Nicholas D. Jeffery, Sarah A. Hamer, Ashley B. Saunders

**Affiliations:** 1 Department of Small Animal Clinical Sciences, School of Veterinary Medicine and Biomedical Sciences, Texas A&M University, College Station, TX 77843-4474, USA; 2 Department of Veterinary Clinical Medicine, College of Veterinary Medicine, University of Illinois, Urbana, IL 61802, USA; 3 Department of Veterinary Integrative Biosciences, School of Veterinary Medicine and Biomedical Sciences, Texas A&M University, College Station, TX 77843-4458, USA

**Keywords:** cardiomyopathy, echocardiography, myocarditis, troponin, *Trypanosoma cruzi*

## Abstract

**Simple Summary:**

*Trypanosoma cruzi* infection causes Chagas disease in dogs and people by damaging the heart with inflammation and fibrosis, resulting in heart enlargement, abnormal function, and irregular heart rhythms. Cardiac magnetic resonance imaging can detect damage to the heart in people with Chagas disease even when other diagnostic tests cannot. The objectives of this study were to describe cardiac magnetic resonance imaging in naturally infected, asymptomatic dogs with chronic Chagas disease and the frequency of abnormalities for cardiac magnetic resonance imaging and other diagnostic tests, including cardiac ultrasound, electrocardiography, and the cardiac biomarker troponin I. In 10 chronically infected dogs. Abnormal findings were present most often with cardiac magnetic resonance imaging (seven dogs) followed by ultrasound (six dogs), electrocardiography (four dogs), and troponin (one dog). Cardiac magnetic resonance imaging abnormalities included increased fibrosis and abnormal wall motion of the heart. The results of this study suggest cardiac magnetic resonance imaging can provide useful information in dogs with *T. cruzi* infection and may support naturally infected dogs for future clinical investigation as an animal model for Chagas disease.

**Abstract:**

*Trypanosoma cruzi* infection causes inflammation and fibrosis, resulting in cardiac damage in dogs. The objectives of this study were to describe cardiac magnetic resonance imaging (CMR) in naturally infected dogs with chronic Chagas disease and the frequency of abnormalities for CMR and cardiac diagnostic tests. Ten asymptomatic, client-owned dogs seropositive for *T. cruzi* were prospectively enrolled in an observational study evaluating echocardiography, ECG (standard and ambulatory), cardiac troponin I (cTnI), and CMR. Standard ECG measurements (3/10) and cTnI concentration (1/10) outside the reference range were uncommon. Ambulatory ECG abnormalities were documented more frequently (6/10 dogs) than with standard ECG and included ventricular arrhythmias (4), supraventricular premature beats (3), second-degree atrioventricular block (2), and sinus arrest (1). Echocardiographic abnormalities were documented in 6/10 dogs including mildly increased left ventricular internal dimension in diastole (1) and decreased right ventricular (RV) systolic function based on reductions in tricuspid annular plane systolic excursion (3) and RV S’ (4). Abnormalities were detected with CMR in 7/10 dogs including delayed myocardial enhancement in 5 of which 2 also had increased extracellular volume, abnormal wall motion in 5, and loss of apical compact myocardium in 1. In conclusion, CMR abnormalities were common, and the results of this study suggest CMR can provide useful information in dogs with *T. cruzi* infection and may support naturally infected dogs for future clinical investigation as an animal model for Chagas disease.

## 1. Introduction

*Trypanosoma cruzi* infection is a cause of cardiac disease in dogs [1,2]. Infected dogs can serve as sentinels for the presence of *T. cruzi* infection in humans [3,4]. Cardiac damage induced by infection with *T. cruzi* is characterized by inflammation, necrosis, and replacement fibrosis, with many similarities between humans and dogs [5,6]. Damage to the heart manifests as electrical conduction abnormalities, arrhythmias, myocardial dysfunction, and heart enlargement [2,6,7,8,9]. While an estimated 20–30% of infected humans develop clinical signs of Chagas cardiomyopathy, including sudden death, others remain subclinical for life despite evidence of cardiac damage on diagnostic evaluation [10,11,12]. The percentage of dogs that will develop clinical disease is not well studied but may be similar to that of humans [13]. In a study of mongrel dogs inoculated with *T. cruzi*, 28% exhibited a reduction in ejection fraction over six to nine months [13]. Additionally, 28% (5/18) of inoculated Beagle dogs developed echocardiographic changes including cardiac chamber dilation and wall dyskinesis over 8 to 36 months [1].

In humans, cardiac magnetic resonance imaging (CMR) is a useful tool to detect early myocardial involvement in as many as 20% of seropositive, asymptomatic patients [14,15]. Myocardial changes detected with CMR in chronic Chagas cardiomyopathy include delayed myocardial enhancement (DME), increased extracellular volume (ECV), ventricular systolic dysfunction, wall-motion abnormalities (WMA), and apical aneurysm [14,15,16,17,18,19]. Infected humans with echocardiographic or electrocardiographic abnormalities are more likely to have DME, a marker of myocardial fibrosis [17]. Myocardial fibrosis and scar tissue assessed by DME increase in the more severe stages of Chagas cardiomyopathy and are associated with ventricular tachycardia, WMA, and shorter survival time in humans [17,20]. Compared to transthoracic echocardiography, CMR is a more sensitive method of detecting WMA in humans. Application of CMR in *T. cruzi* infected dogs has not been described.

The objective of this study was to describe abnormal CMR imaging findings in dogs with chronic Chagas disease and the frequency of abnormalities for CMR and routinely available cardiac diagnostic tests including transthoracic echocardiography, electrocardiography, and cardiac troponin I (cTnI). We hypothesized that infected, asymptomatic dogs would have detectable CMR abnormalities.

## 2. Materials and Methods

### 2.1. Patient Selection

This prospective, observational case series was reviewed and approved by the Institutional Animal Care and Use Committee and the clinical research review committees at Texas A&M University (IACUC 2017-0116). Written informed consent was obtained from each owner before admission of dogs into the study.

The study included a convenience sample of client-owned dogs with positive serology for *T. cruzi* by immunofluorescent antibody (IFA) test performed within the previous three months. Dogs were recruited via the teaching hospital website. Dogs were eligible for enrollment if they were considered healthy without any reported clinical signs by the owners and weighed >15 kg. Dogs were excluded if they had a history of cardiovascular disease or had received treatment for *T. cruzi* infection with any of the following medications: nifurtimox, benznidazole, allopurinol, itraconazole, posaconazole, ravuconazole, or other triazoles. Dogs were also excluded if they were considered high risk for anesthesia, CMR, or intravenous contrast administration (i.e., device implant in the heart, elevated kidney values, or clinically important systemic medical disease) [21].

Dogs were evaluated with a complete physical examination; laboratory analysis including complete blood count, serum biochemistry panel, serum cardiac troponin I (cTnI) using a high-sensitivity assay validated in dogs at the Gastrointestinal Laboratory at Texas A&M University (Ultra-TnI, Advia Centaur CP^®^, Siemens Medical Solutions USA, Inc. Malvern, PA, USA) [22], and immunofluorescent antibody (IFA) test for anti-*T. cruzi* IgG antibodies with titers reported (Texas A&M Veterinary Medical Diagnostic Laboratory, College Station, TX, USA); 6-lead ECG; ambulatory ECG (Holter); echocardiogram; and CMR. The CMR study was scheduled the day after the initial evaluation once the Holter monitor recording was completed.

Outcome for each dog was recorded at 1 and 2 years after enrollment, and owners were asked to consider submitting their dog for a necropsy if a dog died during or after the study period.

### 2.2. Electrocardiography

Electrocardiographic evaluation consisted of a 5-min, 6-lead ECG (GE Healthcare CardioSoft V6.73) obtained with dogs gently restrained in right lateral recumbency without sedation or anesthesia. Recorded measurements included average heart rate, duration of ECG waves (P, PR, QRS) in milliseconds (ms), and height (P, R) in millivolts (mV) made in lead II. Ambulatory ECG recordings were obtained by placing Holter monitor systems (Mortara H3+) as previously described [23]. Briefly, an area was shaved and cleaned with alcohol on both sides of the thorax in preparation for electrode placement. Electrodes were placed and secured with tape or a vest, and the ECG was obtained for 24 h at a recording speed of 25 mm/sec using a lead configuration of V1, V2 and V5. Recordings were analyzed (Del Mar Reynolds software, LabCorp, Burlington, NC, USA) and the full disclosure reviewed (D.J.M., A.B.S.) with the following data recorded: duration of analysis, heart rate (average, maximum, minimum), presence and number of ventricular premature complexes, presence and number of supraventricular premature complexes, presence of supraventricular tachycardia, number of pauses over 3 s, longest pause > 4 s, and presence of second or third degree atrioventricular block. Ventricular arrhythmias were assigned a modified Lown score based on the highest grade observed as follows: 1 = single ventricular premature complexes, 2 = ventricular bigeminy or trigeminy, 3 = accelerated idioventricular rhythm, 4 = ventricular couplets or triplets, and 5 = ventricular tachycardia or R-on-T phenomenon [24,25].

### 2.3. Echocardiography

Transthoracic echocardiography was obtained with simultaneous ECG (Vivid E95, GE Healthcare, Horten, Norway) without sedation or anesthesia. Dogs were gently restrained in right and left lateral recumbency and studies were performed by a cardiology resident under direct supervision of a board-certified cardiologist. The echocardiographic studies were reviewed, and measurements were made on a digital workstation (GE EchoPAC v203; GE Medical Systems, Horten, Norway). Each measurement was repeated 3 times on cardiac cycles consisting of sinus beats, and the mean used for further analysis. Measurements included left ventricular internal dimension at end-diastole and end-systole (LVIDd and LVIDs) from M-mode measurements of the left ventricle in a right parasternal short-axis view that were used to calculate fractional shortening ([LVIDd − LVIDs]/LVIDd × 100). Measured values were normalized to body weight (LVIDdN, LVIDsN) [26]. The left atrium to aorta ratio (LA:Ao) was calculated from measurements of the left atrium and aorta obtained in a right parasternal short-axis view [27]. The diameters of the right atrium and left atrium were measured in a right parasternal, long-axis 4-chamber view across the mid-section of each chamber parallel to the mitral or tricuspid annulus one frame before mitral or tricuspid valve opening [28,29]. As an estimate of right atrial enlargement, the RA:LA ratio was calculated as the long-axis diameter of the right atrium to the long-axis diameter of the left atrium. Likewise, from the right parasternal long-axis 4-chamber view, the left and right ventricular internal dimensions in diastole were measured at the level of the chordae tendineae and the RVIDd:LVIDd ratio was calculated as an estimate of right ventricle (RV) enlargement [28,30]. In a left parasternal long-axis 4-chamber view, left ventricular, Simpson’s method of discs measurements were made to obtain left ventricular volume in diastole and systole and to calculate the ejection fraction (EF). Similarly, from this image, left ventricular wall-motion abnormalities were noted if observed on subjective assessment. Images of the left ventricle from a left parasternal four-chamber long axis view were obtained and stored for offline two-dimensional speckle tracking analysis of global longitudinal strain using commercially available software (EchoPac Q analysis, GE Medical Systems, Horten, Norway). In a left parasternal long-axis 4-chamber view, mitral inflow peak E and A wave velocity were obtained using pulse wave Doppler with the sample volume placed at the tips of the mitral valve leaflets. In a left parasternal long-axis view with the RV maximized in the shape of a triangle and without the left ventricular outflow tract in view, M-mode imaging was performed with the cursor placed through the tricuspid valve lateral annulus for measuring tricuspid annular plane excursion (TAPSE) normalized to body weight (TAPSEn) and pulse-wave tissue Doppler imaging derived peak systolic longitudinal myocardial motion velocity (RV S’) indexed to body weight (iRV S’) for assessing indices of right ventricular systolic function [31,32]. Cutoffs for variables were LVIDdN > 1.85 and LVIDsN > 1.26 [26]; FS < 20% [33], EF < 46% [34], LA:Ao > 1.57 [27], RVIDd:LVIDd > 0.5 [35], TAPSEn < 4.77 [36], iRV S’ < 4.3 [36], and global strain < −15% [37]. Concurrent heart disease was recorded if present.

### 2.4. Cardiac Magnetic Resonance Imaging

Dogs were fasted overnight prior to anesthesia for CMR. The anesthesia protocol consisted of premedication with butorphanol 0.2 mg/kg IV, induction with propofol 5 mg/kg IV, and maintenance of anesthesia with isoflurane (0.5–4%) inhalant. Images were acquired with a 32-channel 3T system with phased-array cardiac coils (MAGNETOM Verio, Siemens AG, Healthcare Sector Erlangen, Germany). Scout images were used to identify the long-axis and short-axis views of the LV, as well as the two- and four- chamber views of the heart. ECG-gated Flash cine mode with retrospective gating was used to acquire dynamic cine loops of the heart and stacks of short-axis images from the aortic root to the apex with the following parameters (Slice thickness: 6 mm, no gaps, field of view: 400 mm, TR/TE: 534 ms/1.32 ms, voxel size: 2.8 × 2.1 × 8.0 mm, pulse flip angle: 44–47°). T1 mapping was performed from short-axis planes at the base and mid-left ventricular chamber using an ECG-triggered, modified Look-locker inversion recovery sequence. The T1 maps were acquired before and 15-min after contrast injection using gadobutrol contrast (Gadovist, Bayer HealthCare, Whippany, NJ, USA) administered intravenously at 0.2 mmol/kg. Late gadolinium enhancement images were obtained 10-min after the bolus of gadobutrol using an inversion-recovery gradient echo technique. Inversion time was individually determined based on TI prep pulse sequencing prior to contrast administration and manually adjusted (260–480 ms) to null the myocardium and enhance any areas of contrast uptake. All images were acquired during breath holding at end expiration by turning the respirator off (average, 12–18 s) and stored digitally for off-line analysis.

The studies were independently analyzed by one observer with experience interpreting CMR in dogs who was blinded to all other diagnostic test results (RF). Left and right ventricular volumes were derived, and EF was calculated offline using the semiautomatic software (Argus, Siemens AG, Healthcare Sector Erlangen, Germany) as previously described in normal dogs [38]. The left ventricle was separated into 8 cuts that were divided into 48 segments as follows: apical cuts 1 and 2 with 4 segments each, middle cuts 3 through 6 with 6 segments each, and basal cuts 7 and 8 with 8 segments each. Each segment was visually inspected and scored for DME and a proportion was calculated as the number of segments affected out of the total number of segments.

The presence of WMAs was classified as mild hypokinesis, severe hypokinesis, dyskinesis, or akinesis based on visual inspection of the 48 segments. The location of the WMA was recorded, and a proportion was calculated as the number of segments affected out of the total number of segments. Apical wall thinning or aneurysm was also recorded.

Extracellular volume was quantified based on the following formula:

(1) $$ECV=(1-\mathrm{hematocrit})\frac{\frac{1}{\mathrm{post}\;\mathrm{contrast}\;T1\;\mathrm{myocardium}}-\frac{1}{\mathrm{native}\;T1\;\mathrm{myocardium}}}{\frac{1}{\mathrm{post}\;\mathrm{contrast}\;T1\;\mathrm{blood}}-\frac{1}{\mathrm{native}\;T1\;\mathrm{blood}}}$$

Values over 24% were considered abnormal [39,40].

### 2.5. Statistical Analysis

Sample size calculation was based on reports that CMR can detect early myocardial involvement in as many as 20% of seropositive, asymptomatic human patients with Chagas disease [14,17]. For this study, enrollment of 10 dogs from a population of infected dogs had a 90% chance of identifying CMR lesions in at least 1 dog with 80% power. Descriptive statistics were generated for the 10 dogs. Values were reported as median (range) or number (proportion).

## 3. Results

The ten dogs that met the inclusion criteria were prospectively enrolled between September 2017 and July 2018 including seven females (five spayed, two intact) and three males (all intact). Breeds included English pointer (n = 3), English springer spaniel (n = 2), and one each of Brittany spaniel, German shorthaired pointer, Labrador retriever, pit bull terrier, and standard poodle. The median age was 5.7 years (range, 1.8–10.9 years), and median weight was 19.2 kg (range, 15.4–32.3 kg). Counties of residence were Bastrop (four), Lee (two), and one each for Bexar, Brazos, Coryell, and Live Oak.

No abnormalities were detected on complete blood count and serum biochemistry panel in any dog. The range for IFA titers was from 160 to >1280 (reference < 20) prior to enrollment and from 80 to >1280 at the time of enrollment. Titers were stable over the three-month period based on values that were unchanged (n = 4) or had increased (n = 2) or decreased (n = 4) by no more than a one-fold change. Median cTnI concentration was 0.0675 ng/mL (range, 0.010–0.242 ng/mL; reference range, 0.006–0.128 ng/mL) with one dog above reference range [22].

All dogs were in a sinus rhythm on six-lead ECG, and median heart rate was 126 beats/min (range, from 80 to 145 beats/min). Prolongation of the P wave duration was detected in three dogs and was the only abnormality detected on standard ECG [41]. Measured variables are listed in Table 1. The median analyzed time for ambulatory ECG was 21.1 h (range, from 19.8 to 25.0 h). Median values for heart rate included a maximum rate of 221 beats/min (range, 172–257 beats/min), average rate of 82 beats/min (range, 72–104 beats/min), and minimum rate of 36 beats/min (range, 21–52 beats/min). Ventricular arrhythmias were documented in four dogs with 1, 4, 482, and 2168 total number of ventricular premature beats and modified Lown scores of 1, 1, 3, and 5, respectively. Supraventricular premature beats were documented in three dogs, and second-degree atrioventricular block Mobitz type II in two dogs. Sinus arrest > four s in duration was documented in one dog (4.4 s). Three dogs had more than one abnormality recorded with ambulatory ECG.

Echocardiographic results are summarized in Table 2. Measurements of left atrial size, left ventricular size, and left ventricular systolic and diastolic function were normal except for one dog with mild left ventricular enlargement based on an LVIDdN that exceeded the 95% reference interval. Left ventricular wall-motion abnormalities were not identified subjectively or with global strain assessment. Measurements of right atrial and right ventricular size were normal. Indices of right ventricular systolic function were reduced in three dogs based on TAPSEn and four dogs based on iRV S’. This resulted in five dogs with echocardiographic abnormalities attributed to *T. cruzi* infection. Atrioventricular valve regurgitation was also documented in four dogs and was not attributed to *T. cruzi* infection. Two dogs had mitral regurgitation and thickening of the mitral valve leaflets attributed to myxomatous mitral valve disease with normal heart size and were staged as B1 based on consensus statement guidelines [42]. Three dogs had tricuspid valve regurgitation (2 trace, 1 mild with peak velocity 2.8 m/s) one of which also had mitral valve regurgitation.

**Figure 1:**
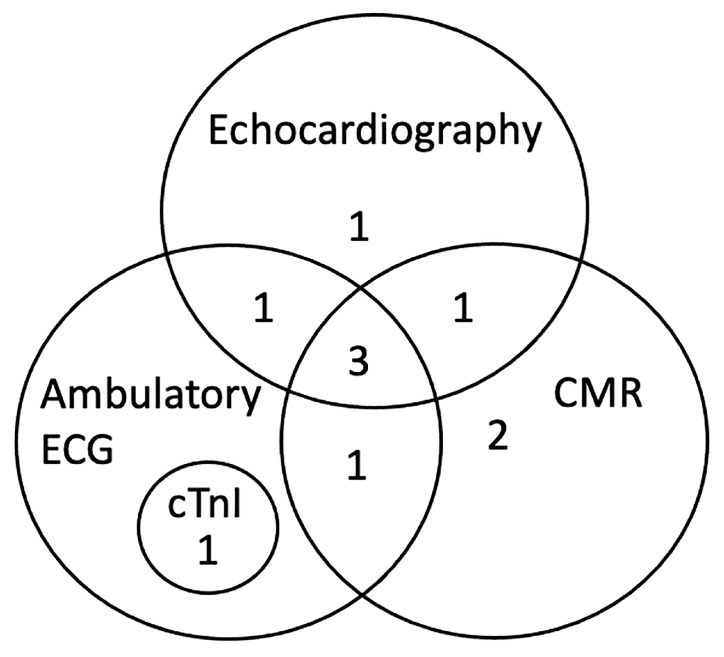
Classification of abnormalities by diagnostic tests including CMR (n = 7), echocardiography (n = 6), ambulatory ECG (n = 6), and cTnI (n = 1) in 10 naturally infected dogs with chronic Chagas disease. CMR, cardiac magnetic resonance imaging; cTnI, cardiac troponin I.

**Figure 2:**
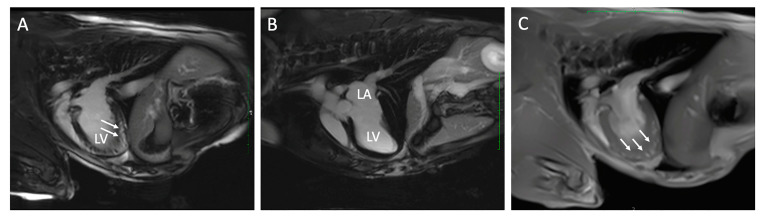
Cardiac magnetic resonance images from two dogs with chronic Chagas disease. In Panel (**A**), delayed myocardial enhancement (arrows) is demonstrated in the left ventricular (LV) apex compared to a dog without DME (Panel (**B**)). Panel (**C**) is a FLASH Cine image three-chamber view in the same dog as panel (**A**) with DME that demonstrates loss of myocardial compaction at the LV apex (arrows). LA, left atrium.

Results from CMR imaging are included in Table 2. The comparative classification of the abnormalities in the 10 dogs is displayed in Figure 1 and categorized for each dog in Table 3. Abnormalities were identified in 7/10 (70%) dogs including DME in 5/10 (50%) and WMA in 5/10 (50%), three of which had both. Median DME in the LV was 6.25% (range, 2.08–16.70%) based on percentage of segments affected. Areas of DME were observed in the interventricular septum (n = 3), left ventricular freewall (n = 2) and a posterior papillary muscle in the left ventricle (n = 1). One dog with diffuse DME of the left ventricular freewall also had loss of the compact myocardium characterized by wall thinning of the apex (Figure 2). Left ventricular ECV fraction was greater than 30% in two dogs with DME. In the five dogs with WMA, a median of 9 of the 48 segments evaluated were affected (range, from 2 to 11 segments). Abnormalities were characterized by dyskinesis of the inferior basilar septum in two dogs and hypokinesis of the anterioinferior midventricular and interventricular basilar septum, basilar anteriolateral septum, and posterior lateral freewall in the mid apical region, posterior and anterior apex and mid left ventricle in five dogs. Two of the seven dogs with DME and WMA on CMR imaging did not have any abnormal findings on echocardiography, cTnI or ambulatory ECG. Ejection fraction for the left and right ventricle were 32.9% (range, 25.8 to 57.0) and 22.5% (range, 15.2 to 52.4) respectively. Because the EF was within reference range when obtained with echocardiography while the dogs were awake [38], the lower CMR EF values were attributed to the effects of anesthesia [43].

All dogs were alive one year after enrollment. At two years, seven dogs were alive and three were lost to follow up. Two dogs were euthanized at 2.1 and 2.3 years after study enrollment unrelated to Chagas disease and necropsies were performed. One was diagnosed with a linear foreign body and the other with liver hepatopathy and acute necrosis. On histopathologic evaluation, protozoal organisms were not identified in any organ from either dog. In one dog (12-year-old, male, Brittany spaniel), gross evaluation of the heart showed an irregular, ill-defined pallor of the right ventricular epicardium near the groove of the interventricular septum which was classified as mild, multifocal chronic lymphoplasmacytic myocarditis with cardiomyocyte loss on histopathology. No abnormalities had been identified with the study CMR. In the second dog (four-year-old, female, standard poodle), gross evaluation of the heart showed a focal flat, pale tan region in the left ventricle adjacent to the interventricular septum and moderate, multifocal, chronic lymphoplasmacytic myocarditis and cardiomyocyte degeneration with interstitial fibrosis and fibrofatty infiltration which was more severe along the interventricular septum. Sampled myocardial tissue was PCR positive with *T. cruzi* strain TcI [44]. Incidentally, six fetuses were found at necropsy and tissue from the fetal hearts and umbilicus were sampled and were PCR negative for *T. cruzi*. Mild hypokinesis of the anterioinferior midventricular septum had been identified with the study CMR. Both dogs had evidence of abnormal RV systolic function based on reduced S’ and otherwise normal echocardiographic evaluations at the time of study enrollment.

## 4. Discussion

In this study, we documented CMR abnormalities in asymptomatic dogs with naturally acquired chronic Chagas disease. In this population of dogs, CMR, echocardiographic, and ambulatory ECG abnormalities were detected most often.

Cardiac magnetic resonance imaging provides a unique high spatial resolution method for detecting abnormal wall-motion, apical thinning, and aneurysms, as well as defining tissue properties including perfusion and fibrosis, and assessing global ventricular function. Fibrosis and scar tissue can be detected ante-mortem with CMR by evaluating DME, calculating ECV, and with T1 mapping [17,20]. In chronic Chagas disease, fibrosis replacing normal cardiomyocytes is a characteristic finding on histopathologic evaluation of the myocardium along with variable inflammation and scarce pseudocysts containing amastigotes [5,6]. In humans, myocardial fibrosis diagnosed by detecting DME is reported in 20% of people with chronic Chagas disease [45], and larger areas of DME are more likely to be associated with reduced LV EF [46]. Fifty percent of the dogs in this study had areas of DME, two of which also had increased ECV.

The most common locations of DME in humans with chronic Chagas disease are at the apex and inferolateral segments of the LV [46]. Fibrosis can be detected in all four cardiac chambers in inoculated dogs in the chronic stage of Chagas disease with most lesions identified in the left ventricle [5]. Dogs in this study had DME in the LV detected most often at the basilar septum and apical portion of the posterolateral freewall. Apical wall thinning and aneurysmal lesions are reported in humans with Chagas disease and can be detected with echocardiography and CMR [47,48,49]. Echocardiographic identification of apical wall thinning has been reported in a dog with complex arrhythmias that did not have CMR performed [50]. One dog in this report had DME and apical wall thinning detected with CMR that was not appreciated with echocardiography.

In 7% of infected humans, DME is identified without ECG or echocardiographic abnormalities [14]. Two of the five dogs with DME in this study did not have abnormalities on the other diagnostic tests performed similar to what has been described in humans [14]. Additionally, CMR is considered a complimentary test for identifying cardiac abnormalities in humans. Our study supports this idea in dogs, in which all 10 dogs had an abnormality detected on at least one diagnostic test performed.

In humans, fibrosis is a predictor of arrhythmia formation in patients with and without normal myocardial function and has been associated with an increased risk of ventricular arrhythmias and ventricular tachycardia [20,45,46]. Areas of fibrosis are associated with ECG conduction abnormalities and the development of re-entry circuits that predispose to ventricular arrhythmias [45,51,52]. Similarly to previous reports, dogs in this study were more likely to have arrhythmias detected with ambulatory ECG than with a standard ECG in which only conduction abnormalities were detected [25,53,54], and the arrhythmias identified were characteristic of chronic Chagas disease ranging from simple to complex. Ventricular arrhythmias were frequently a component of more complex arrhythmias and were identified in dogs with CMR abnormalities (DME or WMA).

Wall-motion abnormalities were detected with CMR in 50% of dogs in this study and were not uniformly distributed, similarly to what is reported in humans [17]. In humans with Chagas disease, WMAs are associated with more severe disease and a worse prognosis [45]. Additionally, the percentage of patients with WMAs increases in the chronic stage of the disease [48]. While WMAs and LV systolic dysfunction were detected with CMR, they were not detected with echocardiography in dogs in this study. Echocardiography provides an assessment of chamber size and function. In this study, chamber size on both the right and left sides of the heart was predominately normal and left ventricular systolic and diastolic function were normal. Abnormal RV systolic function was present based on reductions in TAPSE and RV S’. These measurements of RV systolic function have been evaluated as predictors of outcome in predominately right sided heart diseases in dogs including arrhythmogenic right ventricular cardiomyopathy, pulmonary hypertension, and pulmonary valve stenosis [35,36,55]. Echo derived RV S’ correlates with reduced RV ejection fraction in infected humans, and RV systolic dysfunction is associated with worse prognosis [56]. In dogs, RV enlargement has been associated with a worse prognosis and can be an indicator of end-stage disease [25,57]. Advanced echocardiographic imaging with regional speckle tracking of both ventricles may provide earlier detection of wall-motion changes and myocardial damage [56,58].

Cardiac troponin is a marker of myocardial damage that can be elevated in dogs with myocarditis attributed to *T. cruzi* infection [9,25,53]. Serum cTnI concentrations were predominately normal in this study; this is consistent with the chronic disease status of these asymptomatic dogs, and in the one dog with elevated cTnI concentration, arrhythmias were present on ambulatory ECG, but no abnormalities were detected with CMR.

Historically, predominately inoculated dogs have been used as experimental models of Chagas disease [7,59]. Our study shows that naturally infected dogs have similar abnormalities to humans when evaluated with a comprehensive set of diagnostic tests suggesting a potential use as a model of Chagas disease in future study that would benefit both humans and dogs.

Limitations of this study include the small sample size, absence of a control group and inclusion of only asymptomatic dogs. Despite the sample size, 7/10 (70%) had CMR abnormalities underscoring the frequency of cardiac damage that can occur with this disease. Although there was not a contemporaneous control group, we compared CMR results to apparently healthy dogs reported by our group using a similar CMR protocol [38]. The use of client-owned dogs inhibited the ability to pair CMR findings with contemporaneous gross evaluation and histopathology, and histopathology was limited to those owners willing to contribute. Additionally, one dog with abnormal cardiac pathology did not have abnormalities on CMR. It is possible that mild myocarditis present in this dog was not detectable by CMR or that it developed over time in the more than two-year time frame between CMR and histopathologic evaluation. Dogs in this study were asymptomatic and chronically infected representing a subset of dogs with Chagas disease. Case selection and study design were based on an initial exploration into characterizing CMR abnormalities, and knowledge that the ability to acquire a high-quality CMR imaging study is inhibited by frequent arrhythmias, and anesthesia is complicated by impaired ventricular function. Future evaluation of CMR imaging in dogs with clinical disease may provide additional information. In this study, CMR function, myocardial mapping, and delayed-enhancement images prioritized the LV in both the short- and long- axis views, while the entire RV could only be assessed form the short-axis stacks. Additionally, some dogs lacked either a full basilar or apical slice, limiting interpretation of these areas.

## 5. Conclusions

In conclusion, CMR abnormalities were common in dogs in this study, and the results suggest that CMR can provide useful information for future clinical investigation in dogs with Chagas cardiomyopathy including disease staging and may support naturally infected dogs as an animal model for Chagas disease.

## Figures and Tables

**Table 1:** Electrocardiographic variables reported as median (range) in 10 naturally infected dogs with chronic Chagas disease.

Variable	Value	Reference Range
P duration (ms)	40 (39–60)	<40
PR duration (ms)	120 (70–120)	60–130
QRS duration (ms)	40 (40–60)	<70
P amplitude (mV)	0.2 (0.1–0.3)	<0.4
R amplitude (mV)	1.9 (0.9–2.8)	>0.5

**Table 2:** Echocardiographic and cardiac magnetic resonance (CMR) imaging variables in 10 naturally infected dogs with chronic Chagas disease.

Echocardiographic Variables	Median (Range)
LVIDd (cm)	3.78 (3.07–3.06)
LVIDdN	1.57 (1.34–1.75)
LVIDs (cm)	2.65 (1.96–3.20)
LVIDsN	1.02 (0.80–1.16)
LV FS (%)	30.7 (24.0–46.7)
LVEDV (mL)	49.29 (36.83–67.38)
LVESV (mL)	19.08 (15.68–26.03)
LV EF (%)	59.9 (51.2–65.5)
MV E (m/s)	0.65 (0.48–0.88)
MV E:A	1.17 (1.00–2.85)
LA:Ao short axis	1.30 (1.04–1.4)
LA diameter long axis (cm)	3.52 (3.27–4.20)
RA diameter long axis (cm)	2.68 (1.98–3.18)
RA:LA	0.75 (0.50–0.90)
RVIDd (cm)	1.51 (1.12–1.93)
RVIDd:LVIDd	0.41 (0.30–0.48)
TAPSE (mm)	13.5 (7.8–19.3)
TAPSEn	5.12 (2.78–8.02)
RV S’ (cm/s)	10 (5–14)
iRV S’	4.8 (2.2–7.3)
GLPS_AVE (%)	−17.4 (-15.5 to –24.7)
**CMR Variables**	
LV SV (mL)	20.9 (10.7–30.5)
LV ECV (%)*	24.9 (20.7–31.7)
HCT (%)	43 (38–48)
LV native T1 time (ms) *	1141 (1050–1198)
LV post T1 time (ms) *	513 (487–675)

Abbreviations: E:A, ratio between peak velocity of early and late transmitral flow velocity; EF, ejection fraction; FS, fractional shortening; GLPS_AVE, overall average of global longitudinal strain obtained from three apical long-axis views; LA, left atrium; LA:Ao, left atrium to aorta ratio; LVIDd, left ventricular internal dimension at end-diastole; LVIDdN, left ventricular internal dimension at end-diastole normalized to body weight; LVIDs, left ventricular internal dimension at end-systole; LVIDsN, left ventricular internal dimension at end-systole normalized to body weight; RA, right atrium; RA:LA, right atrium diameter to left atrium diameter ratio; RV, right ventricle/ventricular; RV S’, peak systolic RV myocardial velocity at the lateral tricuspid annulus; iRV S’, peak systolic RV myocardial velocity at the lateral tricuspid annulus indexed to body weight; RVIDd, right ventricular internal dimension at end-diastole; RVIDd:LVIDd, right ventricular internal dimension at end-diastole to left ventricular internal dimension at end-diastole ratio; TAPSE, tricuspid annular plane systolic excursion; and TAPSEn, tricuspid annular plane systolic excursion normalized to body weight. * Obtained in 9 dogs.

**Table 3:** Diagnostic test abnormalities including cardiac magnetic resonance imaging (CMR) in each of the 10 dogs with chronic Chagas disease.

Diagnostic Test	1	2	3	4	5	6	7	8	9	10
Cardiac troponin I								X		
ECG (standard)					X	X			X	
ECG (Ambulatory)	X	X	X				X	X		X
Echocardiogram	X	X	X		X		X		X	
CMR		X	X	X	X	X	X			X

## Data Availability

Project data are available from the Oak Trust (Digital Repository) through Texas A&M University at: https://oaktrust.library.tamu.edu/handle/1969.1/197534.
